# Prescribing pattern of anti-psychotic medications in patients with dementia in Oman: a retrospective observational study

**DOI:** 10.1186/s43045-022-00275-0

**Published:** 2023-01-07

**Authors:** Zainab Al-Rashdi, Tamadhir Al-Mahrouqi, Siham Al-Shamli, Sathiya Panchatcharam, Fatema Al-Busaidi, Reem Al-Afani, Naser Al-Balushi, Hamed Al-Sinawi

**Affiliations:** 1 Psychiatry Residency Training Program, Oman Medical Speciality Board, Muscat, Oman; 2 Ministry of Health, Muscat, Oman; 3 Research and Studies Department, Oman Medical Specialty Board, Muscat, Oman; 4 Medical Internship Training Program, Sultan Qaboos University Hospital, Muscat, Oman; 5 Behavioural Medicine Department, Sultan Qaboos University Hospital, Muscat, Oman

**Keywords:** Antipsychotics, Dementia, Behavioral symptoms, Neuropsychiatric Symptoms, Oman

## Abstract

**Background:**

Aggression, agitation, psychosis, and sleep disturbances are common behavioral symptoms of people with dementia and they can be distressing for both individuals and their carers. Due to their potential side effects, antipsychotic medications are recommended only for severe behavioral and psychological symptoms of dementia (BPSD). This study explores the prevalence, patterns, and associated factors with antipsychotic drug use among patients with dementia attending geriatric psychiatry services at Sultan Qaboos University Hospital (SQUH). Using a retrospective cross-sectional design, this study examines antipsychotic use among elderly patients aged 60 years or older with dementia who attended geriatric psychiatry services from January 2020 to December 2021. The following information was solicited: socio-demographic factors, type and severity of dementia, presence of co-morbid medical or mental illness, the psychotropic medications prescribed, the anti-psychotic medication use, duration of use, and the indication of use were solicited as well. A multivariate logistic regression analysis was conducted.

**Results:**

The total prevalence of anti-psychotic use among elderly patients with dementia was 56.6%, and among them, 59% were prescribed anti-psychotics for more than 2 years. Being female, having non-Alzheimer’s dementia, experiencing severe stages of dementia, and having other medical or mental co-morbid conditions were independent predictors of antipsychotic drug use (odds ratio [OR] =1.85, confidence interval [CI] =1.04–3.30; OR=2.77, C.I. 1.52–5.04; OR=4.47, C.I. 2.18–9.18; and OR=2.54, C.I. 1.11–5.78, respectively).

**Conclusions:**

Antipsychotic medication use is prevalent among elderly patients with dementia in Oman. The results from this study will help the policymakers and psychiatrists in Oman to plan for the use of non-pharmacological strategies as the first line of management for BPSD.

## Background

During the course of dementia, many patients exhibit non-cognitive symptoms and behaviors which are referred to as neuropsychiatric symptoms or behavioral and psychological symptoms of dementia (BPSD), such as aggression, agitation, psychosis, sleep disturbance, appetite changes, and others [1]. Among patients diagnosed with dementia, more than 90% develop at least 1 of these symptoms during their illness [2].

BPSD is distressful for patients and caregivers as well, therefore to improve the quality of life for both patient and caregiver, BPSD should be targeted during the treatment of Dementia. Behavioral management is recommended to be the first line in treating BPSD, but it may not be effective in severe cases. If the non-pharmacological approach fails then antipsychotics can be used, which are usually reserved for severe cases. However, antipsychotic use among the elderly increases the risk of cerebrovascular adverse events, cognitive decline, parkinsonism, gait disturbance, sedation, and pneumonia [3].

Previous studies have demonstrated a clear association between treatment with antipsychotic medications and increased morbidity and mortality in people with dementia [4]. Therefore, the use of antipsychotics is often limited by their side effects profile [5]. Because of the increased risk of cerebrovascular adverse effects, the National Institute for Health and Clinical Excellence (NICE) guideline, recommended that antipsychotic drugs should not be prescribed for patients whose non-cognitive symptoms were of mild-moderate severity [5]. Previous international studies have addressed the pattern of antipsychotic prescriptions in dementia patients. Many studies indicate that around 32% of patients with dementia received antipsychotics [6, 7]. A systemic review conducted in 2016, including 16 meta-analysis studies to evaluate the use of antipsychotics in patients with dementia, concluded that antipsychotics showed modest efficacy in treating psychosis, agitation, and aggression in patients with dementia [3].

Available data indicates that in patients with Alzheimer’s disease (AD): olanzapine, risperidone, and aripiprazole showed modest benefits in the management of psychosis and aggression over a period of 6–12 weeks [8]. However, quetiapine didn’t show a similar benefit. In addition, there is limited evidence available for the use of antipsychotic medications in individuals with non-Alzheimer’s dementia type [9].

According to numerous studies, antipsychotics are frequently prescribed for dementia patients with limited monitoring of their side effects, and they are often prescribed for lengthy periods (more than 6 months). On the other hand, the benefits of antipsychotic treatment for dementia patients over longer periods are still unknown [8–12].

There is a lack of knowledge about the patterns of anti-psychotic prescription in middle eastern communities. In light of this, a study was done to determine the prevalence and patterns associated with antipsychotic use and to establish a set of recommendations for the administration of antipsychotics to dementia patients as well as for their monitoring.

## Methods

### Study design and setting

This was a retrospective cross-sectional study conducted by extracting 24 months of data from the electronic medical records of elderly patients (age more than or equal to 60 years) diagnosed with a major neurocognitive disorder based on the Diagnostic and Statistical Manual of Mental Disorders fifth edition criteria (DSM-5) [13] who are attending in-patient and/or out-patient geriatric psychiatry services at Sultan Qaboos University Hospital (SQUH), a tertiary referral hospital located in Muscat, the capital city and is providing specialist mental health care services.

### Study sample and sampling method

We included all elderly patients (age ≥ 60 years) attending in-patient and or out-patient geriatric psychiatry services at SQUH who met the DSM-5 criteria [13] for the major neurocognitive disorder during the study period. The study sample was recruited using a systemic random sampling (every second patient) approach. Any recruited participant who failed to meet the inclusion criteria was excluded from the study and replaced with the next potential participant.

### Inclusion and exclusion criteria of the study participants

We included elderly patients (age ≥ 60 years), who were diagnosed with a major neurocognitive disorder based on the DSM-5 criteria [13] during the period from January 2020 to December 2021. We excluded patients whose clinical records had missing data, we tried to compensate by collecting the missing data from the treating psychiatrist, and when this is not possible the patient is excluded.

### Data source and collection

Data were extracted from patients’ electronic medical records from January 2020 to December 2021 through the hospital information system department after obtaining ethical approval from Sultan Qaboos University Hospital Research and Ethics Committee (MREC2636). The research protocols and procedures conformed to the guidance of the World Medical Association’s Declaration of Helsinki for ethical human research.

### Outcome measures

The data extracted from patients’ medical records were classified into (1) socio-demographic factors, following information solicited age, gender, marital status, educational level, and region of residence; (2) type and severity of dementia. The type of dementia was classified based on DSM-5 criteria [13] and the severity of dementia was classified into mild, moderate, and severe, based on the dementia classification in the Rockwood Clinical Frailty Scale [14]; (3) presence of co-morbid medical or mental illness based on DSM-5 criteria [13]; (4) The prescribed anti-psychotic, and anti-dementia medications; (5) the anti-psychotic medication name, duration of use was solicited as well.

### Rockwood Clinical Frailty Scale

A frailty assessment tool is useful for assessing individuals with cognitive impairment. These are helpful in guiding decisions about treatment plans, and there are several scales that can be applied, but a straightforward instrument like the Rockwood Clinical Frailty Scale can be a quick method for assessing individuals who have cognitive impairment. It requires the clinician to observe the patient mobilizing and inquire about their routine physical activity and capacity for self-care. The level of frailty is correlated with the degree of dementia [15]. It has three dementia classifications: mild, moderate, and severe. Individuals with mild frailty are correlated with mild dementia, those individuals forget details of recent events, though they still remember the event itself, repeat the same question/story, and experience social withdrawal. Individuals with moderate dementia, have very impaired recent memory, even though they seemingly can remember their past life events well. As with moderate frailty, they can do personal care with prompting. Individuals with severe dementia, as in severe frailty, cannot perform personal care without help. The scale has moderate to good inter-rater reliability [16] and is a validated diagnostic tool to assess frailty in elderly hospital patients and patients in the emergency room [17].

### Co-morbid medical or mental conditions

The co-morbid medical or mental conditions were measured by the presence of at least one medical or mental illness. The participant’s medical records were reviewed for whether they had one or more medical or mental co-morbid conditions, for example, respiratory disorders (such as obstructive sleep apnea, asthma), cardiovascular disorders (for example dyslipidemia, coronary acute syndrome, hypertension), endocrine disorders (for example diabetes, thyroid dysfunction), hematological disorders (for example, iron deficiency anemia), and neurological disorders (for example, stroke). For mental conditions, we reviewed the medical records and documented co-morbid mental illnesses which were diagnosed based on the DSM-5 criteria [13], for example, affective and anxiety disorders.

### Statistical analysis

Collected data were analyzed using IBM SPSS Statistics 27.0 (IBM Corp. Released 2020. IBM SPSS Statistics for Windows, Version 27.0. Armonk, NY: IBM Corp). For descriptive purposes, continuous variables were presented with mean with standard deviation, and categorical variables were presented with frequency and percentages. The chi-square test was used for comparison between groups; multivariate logistic regression analysis was done using enter method, considering the significant factors from univariate and close to the significant factors (up to 0.2 level). A *p*-value of < 0.05 was taken as statistical significance.

## Results

Table 1 shows the demographics and clinical variables distribution, there were 251 patients diagnosed with dementia with a mean age of 74.43 ± 8.62 years, 57% of the cases were women, 70.5% were married and about 23% were widows/widowers. The majority of the cases 58% were from outside of the capital of Oman. About 46% were experiencing a severe stage of dementia and 28% were in the moderate or mild stage of dementia. A total of 56.6% were prescribed anti-psychotics, among them, 59% of them used the anti-psychotics for more than 2 years. Besides anti-psychotics, 67.3% of patients were prescribed cholinesterase inhibitor drugs (chEI).

**Table 1:** Patients’ demographic and clinical variables distribution

Variables	***n*** (%)
**Age group**	
60–65 years	38 (15.1)
66–75 years	103 (41.0)
76–85 years	83 (33.1)
> 85 years	27 (10.8)
Age during prescribing anti-psychotic (mean±sd)	74.43±8.62
**Gender**	
Male	108 (43.0)
Female	143 (57.0)
**Marital status**	
Single	3 (1.2)
Married	177 (70.5)
Divorced	13 (5.2)
Widow/widower	58 (23.1)
**Region**	
Muscat	106 (42.2)
Outside Muscat	145 (57.8)
**Educational level**	
Illiterate	22 (8.8)
High school graduate	4 (1.6)
High school diploma	13 (5.2)
Bachelor’s degree	6 (2.4)
Unknown	206 (82.1)
**Severity of dementia**	
Mild stage	64 (25.5)
Moderate stage	71 (28.3)
Severe stage	116 (46.2)
**Duration of anti-psychotic use**	
No anti-psychotic prescribed	109 (43.4)
Anti-psychotic prescribed	142 (56.6)
<1 year	21 (8.4)
1–2 years	32 (12.7)
>2 years	77 (30.7)
Unknown duration	12 (4.8)
**Anti-dementia agents used, besides anti-psychotic**	
ChEI	169 (67.3)
ChEI + NMDA antagonist	10 (4.0)
NMDA antagonist	11 (4.4)
No anti-dementia agent used	61 (24.3)

*chEI* cholinesterase inhibitor drugs, *NMDA antagonist* N-methyl-D-aspartate receptor antagonists

Table 2 shows the association between demographic, clinical variables, and anti-psychotic use. The following factors were entered into the model, gender, Alzheimer’s status, severity, use of anti-dementia medications, and the presence of comorbid medical conditions. After adjusting for these factors in the model, the following factors remain independent predictors for the use of antipsychotics. The female gender showed higher use compared to the male gender (OR=1.85; 95% C.I. 1.04–3.30, *p*=0.038), non-Alzheimer’s dementia (OR=2.77; 95% C.I. 1.52–5.04, *P*=0.001). Patients with severe dementia stages were likely to use more medications, and those who had comorbid medical or mental conditions showed statistically significant results (OR=4.47, 95% C.I. 2.18–9.18, *P*=0.001 and OR=2.54, 95% C.I. 1.11–5.78, *p*=0.027, respectively).

**Table 2:** Association between demographic, clinical variables, and anti-psychotic use

Variables	Unadjusted analysis			Adjusted analysis	
	No (***n***=109)	Yes (***n***=130)	***p***-value	OR (95% CI)	***p***-value
**Age group**					
60–65 years	19 (17.4)	18 (13.8)	0.628		
66–75 years	42 (38.5)	58 (44.6)			
76–85 years	35 (32.1)	43 (33.1)			
> 85 years	13 (11.9)	11 (8.5)			
**Gender**					
Male	51 (46.8)	49 (37.7)	0.188	1.0	
Female	58 (53.2)	81 (62.3)		1.85 (1.04–3.30)	0.038
**Marital status**					
Married	80 (74.8)	89 (69.0)	0.612		
Divorced	5 (4.7)	8 (6.2)			
Widow/widower	22 (20.6)	32 (24.8)			
**Dementia type**					
Alzheimer’s	80 (74.1)	66 (52.0)	<0.001		
LBD*	5 (4.6)	9 (7.1)			
Mixed dementia	9 (8.3)	24 (18.9)			
Vascular dementia	14 (13.0)	17 (13.4)			
Others	-	11 (8.7)			
**Alzheimer’s dementia**					
Non-Alzheimer’s Dementia	80 (74.1)	66 (52.0)	<0.001	1.0	
	28 (25.9)	61 (48.0)		2.77 (1.52–5.04)	<0.001
**Severity of dementia**					
Mild stage	38 (34.9)	21 (16.2)	<0.001	1.0	
Moderate stage	34 (31.2)	33 (25.4)		2.01 (0.93–4.37)	0.076
Severe stage	37 (33.9)	76 (58.5)		4.47 (2.18–9.18)	<0.001
**Use of Anti-dementia**					
No	98 (89.9)	104 (80.0)	0.047		
Yes	11 (10.1)	26 (20.0)			
**Comorbid medical or mental conditions**					
No	98 (89.9)	104 (80.0)	0.047	1.0	
Yes	11 (10.1)	26 (20.0)		2.54 (1.11–5.78)	0.027

*LBD* Lewy body dementia

## Discussion

Numerous research looked into how anti-psychotics were prescribed to dementia patients in western societies. And to the best of our knowledge, this is the first investigation into the antipsychotic prescribing patterns among dementia patients in Oman and the Arabian Peninsula.

This study revealed that 56% of patients diagnosed with dementia were prescribed antipsychotics for BPSD, this prevalence is significantly higher compared to that reported in Japan at a prevalence of 10.7 % [18], and in the USA at a prevalence of 14.9% [19]. Moreover, in a recent study conducted among patients with dementia during the COVID-19 pandemic in England showed a prevalence around of 9% [20] Compared to the worldwide prevalence, our study population had a high rate of antipsychotic prescriptions. This could be explained by the following factors: first, our study was conducted at the geriatric psychiatry services at SQUH, a tertiary care hospital, receiving referrals from other primary and secondary care hospitals for severe and complex cases needing specialized management. Second, our facility has almost no resources for alternative non-pharmacological strategies for BPSD management.

Our study found that antipsychotic medications were prescribed more frequently for dementias other than Alzheimer’s. Despite the scant evidence supporting the effectiveness of antipsychotic medications in this population and their potential for harm. The higher prevalence of behavioral symptoms and psychosis in non-Alzheimer’s dementia could be a possible explanation. This finding is consistent with that of a sizable US database study, which found that individuals with Parkinson’s disease and dementia received antipsychotic medications much more frequently than those with other types of dementia [21]. Moreover, results from our study revealed that patients with severe dementia were more frequently prescribed antipsychotics compared to patients with mild dementia. This finding is consistent with results from a systemic review and a meta-analysis showing an increasing prevalence of antipsychotic use with increasing severity of dementia the results revealed that in studies with moderate dementia, the prevalence of antipsychotic use was 12.2%, and in studies with severe dementia, the prevalence was 45.1% [22].

In our study, women were more likely than men to receive an antipsychotic prescription; nevertheless, the relationship between antipsychotic use and gender is still controversial, with some studies reporting that women received antipsychotic prescriptions more frequently than men [23, 24]. Contrary to several other studies’ findings suggesting that men are more likely to receive anti-psychotic prescriptions compared to women [25, 26].

This study showed that there was a greater incidence of antipsychotic prescription when there was co-morbid medical and mental illness. However, a number of recent studies revealed that antipsychotics were less frequently prescribed to dementia patients who had a history of cardiovascular or cerebrovascular conditions such as hypertension, diabetes, stroke, and renal/hepatic dysfunction [27]. Antipsychotics should only be prescribed to individuals with cardiovascular or cerebrovascular illnesses who are extremely distressed and for brief periods of time [5]. Our findings, therefore, indicate that this older population with co-morbid medical and mental illness should be given more attention in the assessment of these individuals' risks and benefits against the antipsychotic prescription.

The results revealed that antipsychotics were used for more than 2 years in most of the cases 30%, and in only 8% of cases antipsychotics were used for less than 1 year. The duration of antipsychotic prescription observed in our study is inconsistent with that found in the UK mental health services [9]. Moreover, the advantages of using antipsychotics for BPSD treatment for longer than 12 weeks are still unknown. Therefore, it is advised to regularly and frequently reevaluate patients to determine whether they still require anti-psychotic treatment [9]..

## Limitation

Data were extracted from the patient’s clinical records; therefore, some errors are expected in data collection. However, we tried to compensate for this by asking the treating psychiatrist for any unclear documented information. Moreover, our study was conducted at a tertiary care hospital, where severe cases are seen, this might have affected the generalizability of our results.

## Conclusions

Identifying the factors that increase the likelihood of antipsychotic prescription may help to consider alternatives to antipsychotic medications at earlier stages of dementia disease. Psychiatrists are advised to use non-pharmacological strategies as a first line in managing BPSD. When nonpharmacologic methods are not effective in treating distressing symptoms then psychiatrists are advised to use anti-psychotic medications, provided that they document in patient clinical notes the indication and the target symptoms for initiating the antipsychotic medication. Moreover, psychiatrists are recommended to perform clinical re-assessment and medications review at least every 6 months, to consider withdrawing the anti-psychotics depending on the clinical assessment. Future studies in Oman could also examine and report polypharmacy, drug-to-drug interactions, and any adverse consequences of antipsychotic treatment in dementia patients. Future studies may also examine the effectiveness of other alternative treatments to antipsychotic medications. Probably, by assessing dementia patients’ quality of life while using non-pharmacological methods and contrasting it with the use of antipsychotic medications.

## Data Availability

The datasets used and/or analyzed during the current study are available from the corresponding author upon reasonable request.

## Abbreviations

BPSD: Behavioral and psychological symptoms of dementia
chEI: Cholinesterase inhibitor drugs
CI: Confidence interval
DSM-5: Diagnostic and Statistical Manual of Mental Disorders Fifth Edition
LBD: Lewy body dementia
NICE: National Institute for Health and Clinical Excellence
NMDA: N-Methyl-D-aspartate receptor antagonists
OR: Odds ratio
SQUH: Sultan Qaboos University Hospital
